# A randomized feasibility trial of the modified Atkins diet in older adults with mild cognitive impairment due to Alzheimer’s disease

**DOI:** 10.3389/fendo.2024.1182519

**Published:** 2024-03-04

**Authors:** Alison Buchholz, Pragney Deme, Joshua F. Betz, Jason Brandt, Norman Haughey, Mackenzie C. Cervenka

**Affiliations:** ^1^ Department of Psychiatry & Behavioral Sciences, The Johns Hopkins University School of Medicine, Baltimore, MD, United States; ^2^ Department of Neurology, The Johns Hopkins University School of Medicine, Baltimore, MD, United States; ^3^ Department of Biostatistics, The Johns Hopkins Bloomberg School of Public Health, Baltimore, MD, United States

**Keywords:** mild cognitive impairment, Alzheimer’s disease, lipidomic, metabolomic, ketogenic, ketone, modified Atkins diet

## Abstract

**Background:**

Alzheimer’s disease (AD) is increasing in prevalence, but effective treatments for its cognitive impairment remain severely limited. This study investigates the impact of ketone body production through dietary manipulation on memory in persons with mild cognitive impairment due to early AD and explores potential mechanisms of action.

**Methods:**

We conducted a 12-week, parallel-group, controlled feasibility trial of a ketogenic diet, the modified Atkins diet (MAD), compared to a control diet in patients with cognitive impairments attributed to AD. We administered neuropsychological assessments, including memory tests, and collected blood samples at baseline and after 12 weeks of intervention. We performed untargeted lipidomic and targeted metabolomic analyses on plasma samples to detect changes over time.

**Results:**

A total of 839 individuals were screened to yield 38 randomized participants, with 20 assigned to receive MAD and 18 assigned to receive a control diet. Due to attrition, only 13 in the MAD arm and nine in the control arm were assessed for the primary endpoint, with two participants meeting ketosis levels used to define MAD adherence criteria. The average change from baseline in the Memory Composite Score was 1.37 (95% CI: −0.87, 4.90) points higher in the MAD group compared to the control group. The effect size of the intervention on baseline MAD change was moderate (Cohen’s *D* = 0.57, 95% CI: −0.67, 1.33). In the 15 participants (nine MAD, six control) assessed for lipidomic and metabolomic-lipidomics and metabolomics, 13 metabolites and 10 lipids showed significant changes from baseline to 12 weeks, including triacylglycerols (TAGs, 50:5, 52:5, and 52:6), sphingomyelins (SM, 44:3, 46:0, 46:3, and 48:1), acetoacetate, fatty acylcarnitines, glycerol-3-phosphate, and hydroxy fatty acids.

**Conclusions:**

Attrition was greatest between baseline and week 6. All participants retained at week 6 completed the study. Despite low rates of adherence by criteria defined *a priori*, lipidomic and metabolomic analyses indicate significant changes from baseline in circulating lipids and metabolites between MAD and control participants at 12-week postrandomization, and MAD participants showed greater, albeit nonsignificant, improvement in memory.

## 1 Introduction

The prevalence of Alzheimer’s disease (AD) is increasing as the aging population grows. In addition to those with dementia, there are about five million persons in the USA with mild cognitive impairment (MCI), likely due to AD (1). Advances in drug therapies for AD have been made (2–4), but concerns have been raised regarding their safety, cost, and translation to clinical outcomes (5, 6). Alternative approaches for the treatment and prevention of AD are needed. Studies examining the influence of dietary changes on cognition and/or functioning in AD have produced promising results (7–9).

The role of lipid modulation in the pathogenesis of AD is under investigation. High serum cholesterol is associated with an increased risk of developing AD (10–17), and older persons who consume a low-cholesterol Mediterranean diet have a lower risk of developing AD (18–20). In mouse models of AD, a high-fat and high-cholesterol diet increases the production of amyloid-β, worsens synaptic damage, and further impairs learning and memory compared with mice fed standard diets (21, 22). As such, the Dietary Approaches to Stop Hypertension (DASH) and Mediterranean-DASH Intervention for Neurodegenerative Delay (MIND) diets have been among the most popular recommended diets for aging people (23, 24).

Ketone bodies produced by the metabolism of fatty acids in the liver are used by cells throughout the body, including the brain, as a source of energy (25). AD brains can metabolize ketone bodies even when glucose metabolism is impaired (26, 27). Given the clearly impaired glucose metabolism in AD (25, 28–34), increased attention has been given to the use of ketogenic diets as treatments for AD.

The ketogenic diet has proven to be an efficacious intervention for drug-resistant epilepsy but requires precise measurement of all macronutrient intake and strict adherence (35–37). Fortunately, ketosis can be induced using a more forgiving diet that is high in fat and low in carbohydrates, such as the modified Atkins diet (MAD), which is also effective in reducing epileptic seizures (35, 38, 39). The MAD is now being investigated as an intervention for several other neurologic conditions (40) and medical diseases such as obesity (41) and type 2 diabetes (42).

Studies examining the use of ketogenic diets in AD generally support the notion that ketogenic diets can enhance cognition in older adults with AD (43–47) and suggest that the degree of cognitive enhancement depends on the level and duration of ketosis, but results have been mixed. Petersson et al. (48) showed that older persons who typically consumed high carbohydrate diets had an increased risk of MCI and dementia (hazard ratio [HR] = 1.89), whereas those with high fat (HR = 0.56) and high protein (HR = 0.79) diets had decreased risks. Krikorian et al. (49) showed that among persons with MCI, only those on low-carbohydrate diets for 6 weeks showed improved memory, which was correlated with urine ketone levels. Taylor et al. (50) demonstrated improved cognition in persons with AD after 3 months on a ketogenic diet and taking a medium-chain-triglyceride (MCT) fat supplement (51). Phillips showed that AD patients on a ketogenic diet improved significantly in quality of life and daily functioning compared to those following “health-eating” guidelines over a 12-week period, but improvements in cognition were not significant (52). Ketosis may not be the only factor influencing memory performance in individuals on ketogenic diet therapy, and further investigation is warranted to examine other potential mechanisms of action.

The present study examined the effects of a 12-week MAD diet on cognition, dietary adherence, ketosis, and lipidomic-metabolomics in seniors with AD-related MCI or mild dementia.

## 2 Methods

We conducted a 12-week, parallel-group, 1:1 randomized controlled trial of the MAD compared to a control diet (based on the National Institute on Aging [NIA]’s dietary recommendations for seniors) for patients with MCI attributed to AD. The study was fully reviewed and approved by the Institutional Review Board of the Johns Hopkins University School of Medicine.

### 2.1 Participants

Complete eligibility criteria, procedures (including detailed dietary information), methods, and clinical outcomes are described in a prior publication (53). Briefly, patients with MCI or early-stage AD were recruited via the Johns Hopkins Alzheimer’s Disease Research Center registry, posted flyers, radio and newspaper advertisements, mailings to persons on the Central Maryland chapter of the Alzheimer’s Association list, and lectures at senior centers and retirement communities.

Patients were required to cohabitate with a cognitively intact study partner whose role was to help the patient shop for and prepare appropriate foods, document food intake, test and record urine ketone levels, attend study visits, and provide information regarding the patient’s functional abilities. Patients taking medications for AD were not excluded from the study, as long as they were on the same dose for 3 months or more before the study began and did not change their dose during the study. Complete eligibility criteria appear in **Supplementary Table S1**.

### 2.2 Procedures

#### 2.2.1 Eligibility screening

Potential participants were contacted by email or telephone to assess their interest and eligibility. In-person screening visits were conducted with the participant and study partner. The in-person screening included administration of the Montreal Cognitive Assessment (MoCA) (54) to study participants, the Mini-Mental State Examination, second edition (Standard Version; MMSE-2) (55) to study partners, and the Clinical Dementia Rating Scale (CDR) (56) to participant and partner. Study partners were required to earn a *T*-score of ≥ 40 (adjusted for age and education) on the MMSE-2 to demonstrate intact cognition. Eligible dyads were taught how to complete food records and provided copies of informed consent forms for their review.

#### 2.2.2 Enrollment/baseline assessments

At the enrollment/baseline visit, informed consent was obtained from the patient and the study partner (dyad). A urine sample, fasting blood sample, and vital signs were procured from the patient. The urine sample was tested for the presence of ketones, participants were offered a light snack, and a dietitian reviewed food records with the dyad.

Neuropsychological testing was performed by a research assistant who was blinded to treatment. Testing included the Mini-Mental State Examination-2 Expanded Version (MMSE-2-EV) (55), the Hopkins Verbal Learning Test—Revised (HVLT-R) (57), and the Brief Visuospatial Memory Test—Revised (BVMT-R) (58). MMSE-2-EV scores range from 0 to 90, with 90 indicating perfect performance. HVLT-R and BVMT-R delayed recall trial scores each range from 0 to 12, with 12 indicating perfect performance.

Finally, dyads who enrolled in the study were randomly assigned to one of two diets, either the MAD or the NIA, by using a random number table. Specifically, after informed consent and baseline data were obtained, the study coordinator accessed the random number generator, and if an even number was next on the list, the dyad was randomized to the MAD, and if an odd number, they were randomized to the NIA. The dyad was given their respective diet manual.

#### 2.2.3 Educational visit

Dyads met with a dietitian and were provided with information to initiate and adhere to their diet, including instructions regarding food label reading and portion measuring, as well as sample menus and recipes. Dyads were taught how to record food intake and perform urine ketone testing. They were given a urine ketone test kit, multivitamins, and calcium with vitamin D supplements. Participants had equal time with and access to study dietitians, regardless of group assignment.

The MAD (59) includes consumption of ¾ 20 or fewer grams daily of net carbohydrates (total carbohydrates minus fiber), large amounts of fat, moderate amounts of protein, and proper hydration. The NIA diet encourages the consumption of fruits, vegetables, beans, peas, nuts, seeds, whole grains, fat-free or low-fat dairy products, seafood, lean meats, poultry, eggs, and oils. The NIA diet limits added sugars, refined grains, sodium, and solid fats.

#### 2.2.4 Follow-up visits

Follow-up assessments, including measurement of vital signs and urine ketone levels, review of food records, and discussion with dieticians of any issues with diets or adherence, were conducted every three weeks. Neuropsychological testing was repeated at weeks 6 and 12, and blood was collected again at week 12. At study visits, dyads were offered avocados, a parking voucher, and a $25 grocery store gift card (totaling $100 across study visits).

### 2.3 Materials

All chemicals and solvents were ultra-pure LC-MS grade (60, 61).

#### 2.3.1 Untargeted lipidomics by MS/MS^ALL^ triple TOF

Plasma samples were extracted using a modified Bligh and Dyer’s procedure (60, 62) to obtain a crude lipid fraction. In brief, 40 μL of each plasma sample was extracted for total lipids using 2 mL of ddH_2_O and 3.8 mL of methanol/dichloromethane (2:1.9, v/v), containing 12 internal standards, as reported earlier (60). Lipid analysis was conducted in MS/MS^ALL^ mode on a TripleTOF 5600 (AB Sciex, Redwood City, CA, USA) time-of-flight mass spectrometer (TOF MS) (60). Samples (50 μL of injection volume) were directly infused by HPLC at a constant flow rate of 7 µL/min. The obtained mass spectral data were processed using the LipidView database (version 1.3, Ab Sciex, Concord, Ontario, Canada) as described (60).

#### 2.3.2 Targeted metabolomics by LC-MS/MS

Metabolites from plasma were extracted as described earlier (61). Briefly, plasma samples (150 µL) were protein precipitated using 1 mL of 70% ice-cold methanol (0.5% 1 N HCl), prespiked with 11 isotopically labeled IS (61). Chromatographic separation of metabolites was achieved on the pentafluorophenyl column (pursuit PFP, 150 mm × 2 mm, 3 µM particle size, Agilent Technologies, CA, USA). A quadrupole ion trap mass spectrometer (API4000 QTRAP LC-MS/MS, AB Sciex, ON, Canada) was used to identify metabolites in pseudo-MRM mode. The mass spectrometer was operated in electrospray ionization (ESI)-positive and ESI-negative modes individually to detect protonated [M+H]^+^ and deprotonated [M-H]**^−^** metabolites. Instrument control and quantification were performed using Analyst 1.4.2 and MultiQuant software (AB Sciex, Thornhill, ON, Canada). The complete list of targeted metabolites and their mass spectrometric details are provided in the **Supplementary Materials**.

### 2.4 Data analysis

Adherence for the MAD participants was determined *a priori* as demonstrating at least moderate urine ketone levels at three or more follow-up visits. The primary outcome for memory was defined as change from baseline to 12 weeks in the Memory Composite Score (MCS), defined as the sum of the delayed recall trials for the Hopkins Verbal Learning Test-Revised (HVLT-R) (63) (a measure of auditory-verbal learning and memory) and the Brief Visuospatial Memory Test-Revised (BVMT-R) (64) (a measure of visuospatial learning and memory).

The average treatment effect of the intervention was assessed using an unadjusted difference in means as well as a covariate-adjusted difference in means. Covariate adjustment was performed using analysis of covariance (ANCOVA), regressing the 12-week outcome on the baseline MCS and treatment assignment. Standardized effect sizes (Cohen’s *D*) were also computed. Confidence intervals and hypothesis tests were conducted using the 10,000 replicates of the nonparametric bootstrap using the bias-corrected and accelerated method. Missing values of covariates were imputed using mean imputation in each bootstrap sample. In analyses of cognitive outcomes, missing follow-up outcome data were addressed using inverse probability weighting (IPW) based on treatment assignment and baseline MCS. Analyses were conducted according to the allocated treatment arm, irrespective of adherence to the intervention; all randomized participants were included in analyses of cognitive outcomes.

Lipidomic and metabolomic data were analyzed using ANCOVA, where each biomarker at 12-week follow-up was regressed on treatment assignment and the biomarker assessed at baseline. Due to sample size limitations, IPW was not performed. Model-based standard errors were used to compute confidence intervals. All biomarkers were log-transformed prior to analysis. Adjustment for multiple testing was performed using the method of Benjamini and Hochberg (65).

All analyses were conducted in R version 4.3.1 (R Foundation for Statistical Computing, Vienna, Austria).

## 3 Results

**Figure 1:**
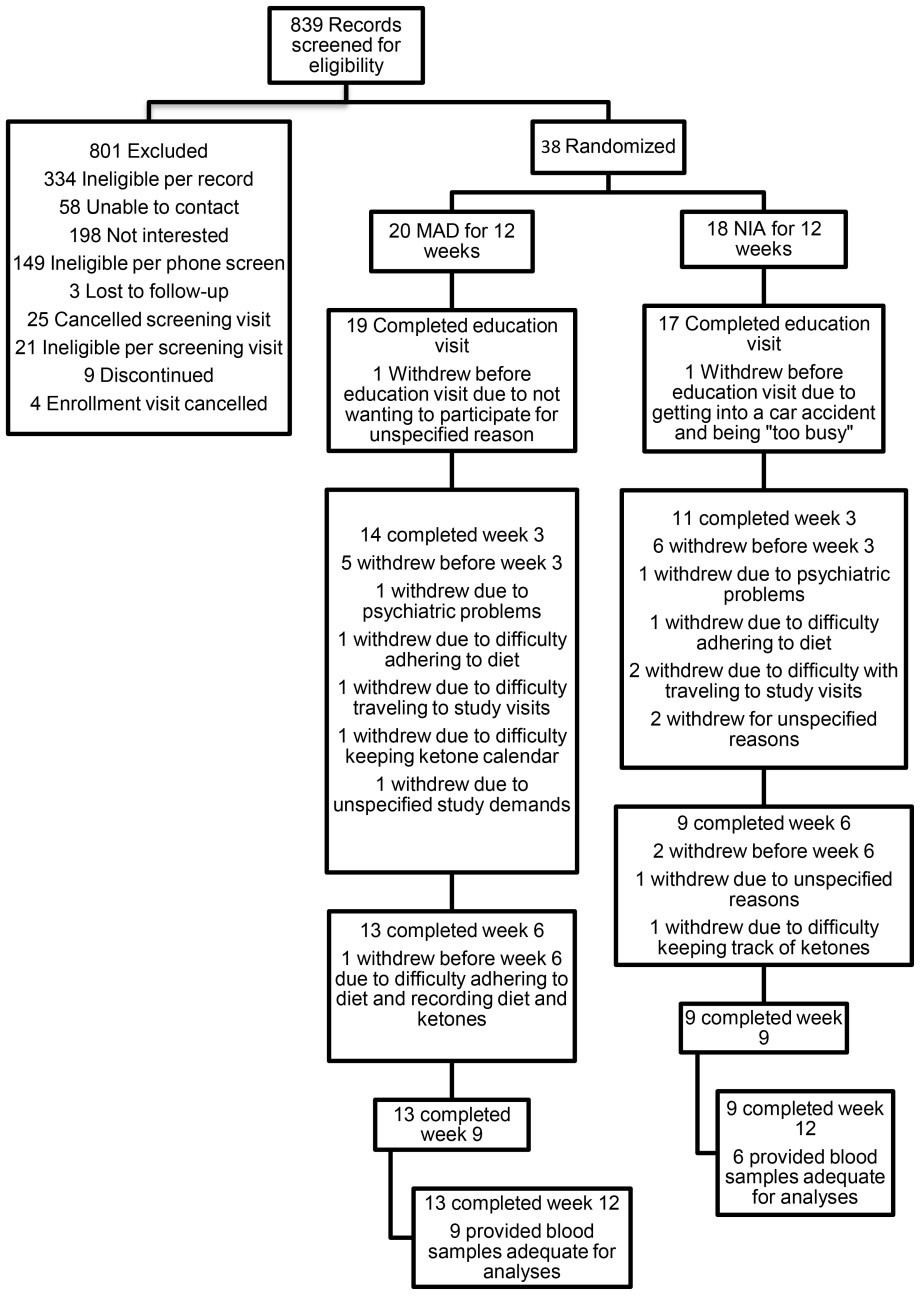
Flow diagram.

As shown in **Figure 1**, 839 people were screened for the larger study from which the data for the present study were extracted (53, 66). The most common reasons for exclusion from the study were medical conditions, severe cognitive impairment, and living alone. While most eligible parties expressed interest in participating in the study, many declined due to not wanting to change their diet, as well as time and travel costs. Ultimately, 20 were enrolled and randomized to the MAD arm, while 18 were enrolled and randomized to the NIA arm. Please see **Table 1** for baseline demographic and clinical factors for all enrolled and randomized participants. In total, 13 of the MAD and nine of the NIA participants completed the study, with only two MAD participants meeting adherence criteria. The most common reasons for withdrawing were no longer wanting to adhere to a diet, as well as time and travel costs. Of the completers, nine of the MAD and six of the NIA were able to provide blood samples deemed adequate for the analyses conducted in the present study; several participants were not able to produce enough blood, and several were not fasting. See **Supplementary Table S2** for demographic information regarding the 15 participants included in lipidomic and metabolomic analyses. There were no adverse events.

**Table 1:** Baseline demographic and clinical factors.

	NIA (*N* = 18)	MAD (*N* = 20)	Overall (*N* = 38)
Age (years)			
Mean (SD)	74.2 (5.8)	74.2 (6)	74.2 (5.8)
Range	[60.9, 83.9]	[64.9, 87.1]	[60.9, 87.1]
Sex			
Male	7 (38.9%)	13 (65.0%)	20 (52.6%)
Female	11 (61.1%)	7 (35.0%)	18 (47.4%)
Race			
White	14 (77.8%)	19 (95.0%)	33 (86.8%)
Non-white	4 (22.2%)	1 (5.0%)	5 (13.2%)
Education (years)			
Mean (SD)	15.9 (3.5)	16.1 (2.2)	16 (2.8)
Range	[9, 20]	[13, 20]	[9, 20]
BMI			
Mean (SD)	26.2 (4.5)	28.1 (5.1)	27.2 (4.8)
Range	[17, 39.7]	[18.6, 39.9]	[17, 39.9]
MCS (HVLT + BVMT delayed)			
Mean (SD)	3.1 (3.9)	5 (5.6)	4.1 (4.9)
Range	[0, 12]	[0, 17]	[0, 17]
Missing	1 (5.6%)	0 (0%)	1 (2.6%)
CDR global score			
0.5	15 (83.3%)	17 (85.0%)	32 (84.2%)
1	3 (16.7%)	3 (15.0%)	6 (15.8%)
MMSE-2 EV			
Mean (SD)	40.1 (12.4)	41.9 (9.5)	41.1 (10.8)
Range	[17, 56]	[25, 62]	[17, 62]
Missing	1 (5.6%)	0 (0%)	1 (2.6%)

The NIA participant missing baseline MCS and MMSE-2 EV withdrew between education and week 3 due to difficulty adhering to the diet.

### 3.1 Memory changes in MAD and control groups

The results of the 12-week baseline change in MCS analyses can be found in **Table 2**. Unadjusted and covariate-adjusted analyses were quantitatively similar, with MAD participants having baseline changes in MCS scores that were on average 1.37 points higher than NIA participants (95% CI: −0.87, 4.90). The estimate of the effect size on baseline change was of “medium” magnitude (Cohen’s *D* = 0.57, 95% CI: −0.67, 1.33) per Cohen’s (1998) criteria, with a confidence interval spanning medium magnitude negative effects to “very large” positive effects.

**Table 2:** Results of 6- and 12-week baseline change in MCS.

	NIA (*N* = 18)	MAD (*N* = 20)
MCS (HVLT + BVMT delayed): W6		
Mean (SD)	2.9 (3.4)	6.6 (7.4)
Range	[0, 11]	[0, 23]
Missing	9 (50.0%)	8 (40.0%)
MCS (HVLT + BVMT delayed): W12		
Mean (SD)	2.4 (3.9)	6.8 (7.6)
Range	[0, 12]	[0, 20]
Missing	9 (50.0%)	7 (35.0%)
MCS change (HVLT + BVMT delayed): W6 − BL		
Mean (SD)	−0.3 (3.9)	1.2 (2.4)
Range	[−10, 4]	[−2, 7]
Missing	9 (50.0%)	8 (40.0%)
MCS change (HVLT + BVMT delayed): W12 − BL		
Mean (SD)	−0.8 (3.8)	0.9 (2)
Range	[−10, 3]	[−2, 4]
Missing	9 (50.0%)	7 (35.0%)

Nine data points are “missing” for the NIA group, given that only half of those enrolled/randomized (nine of 18) completed the study. Eight data points are “missing” for the MAD group given only 12/20 of those enrolled/randomized completed the study. One additional patient in the MAD group did not complete the HVLT and BVMT needed for MCS at week 6, resulting in an additional “missing” data point at week 6.

### 3.2 Lipids and metabolites with MAD and control groups

We have identified a shared presence of 378 lipid species and 68 metabolites in both the control diet and MAD groups. The comprehensive list of these commonly found lipids and metabolites, along with their respective group classifications, can be found in the **Supplementary Materials**. Individuals subjected to a MAD for 12 weeks displayed notable differences in plasma levels of lipids and metabolites when compared to those on the control diet. Specifically, the MAD resulted in lower plasma levels of various lipids, encompassing three triacylglycerols (TAGs, 50:5, 52:5, and 52:6), four sphingomyelins (SM, 44:3, 46:3, 46:0, and 48:1), two lysophosphatidylcholines (LysoPCs, 18:0 and 18:2), two fatty acylcarnitines (hexanoylcarnitine and stearoylcarnitine), a fatty acid (hexanoic acid), a hexose sugar (fructose), and a phosphatidylserine ether species (PS O-40:0). Conversely, individuals on MAD exhibited higher levels of a ketone body (acetoacetate), glycerol-3-phosphate, two hydroxy fatty acids (3-hydroxyoctanoate and 5-hydroxyhexanoate), two phosphatidylethanolamines (PE O-34:1 and PE O-36:3), and choline phosphate. These findings strongly indicate alterations in the circulating lipids and metabolites of individuals following a ketogenic diet therapy in comparison to those on a control diet. The modified list of changes in the lipids and metabolites, including adjusted *p*-values and fold-change values, can be found in **Table 3**.

**Table 3:** The list of lipid and metabolite species that were differentially regulated by the MAD diet compared to the control diet.

Category	Group	Lipids and metabolites	Fold_change_estimate	Fold_change_p_adjust_all
Lipid	Lysophospholipids	LysoPC 18:2	−10.96616371	0.043907247
Metabolite	Fatty acylcarnitines	Hexanoylcarnitine	−5.433043215	0.014075101
Metabolite	Fatty acylcarnitines	Stearoylcarnitine	−4.357305955	0.010114821
Metabolite	Fatty acids	Hexanoic acid	−4.275752368	0.000550305
Metabolite	Sugars	Fructose	−2.228621667	0.034695265
Lipid	Lysophospholipids	LysoPC 18:0	−2.160600267	0.010114821
Lipid	Sphingomyelins	SM 48:2	−1.919390073	0.036361039
Lipid	Triacylglycerols	TAG 52:6	−1.735061488	0.028925726
Lipid	Triacylglycerols	TAG 52:5	−1.72170821	0.041874467
Lipid	Triacylglycerols	TAG 50:5	−1.567460165	0.033099035
Lipid	Sphingomyelins	SM 46:3	−1.563999943	0.021239213
Lipid	Sphingomyelins	SM 46:0	−1.559294415	0.021239213
Lipid	Sphingomyelins	SM 44:3	−1.386330786	0.030309054
Lipid	Phosphatidylserines	PS O-40:0	−1.063554114	0.033099035
Lipid	Sphingomyelins	SM 48:1	−0.838412193	0.025356407
Lipid	Phosphatidylethanolamines	PE O-34:1	0.618222887	0.033099035
Lipid	Phosphatidylethanolamines	PE O-36:3	0.944495468	0.030309054
Metabolite	Ketone bodies	Acetoacetate	1.652590845	0.001410745
Metabolite	Others	Choline phosphate	2.410712721	0.010114821
Metabolite	Hydroxy fatty acid	3-Hydroxyoctanoate	2.433853804	0.025356407
Metabolite	Others	Glycerol 3-phosphate	3.061953992	0.030309054
Metabolite	Hydroxy fatty acid	5-Hydroxyhexanoate	4.096819308	0.000182016

Furthermore, our investigation revealed the presence of 77 distinct lipids that were exclusively identified in either the control or MAD groups. Notably, a significant observation was that several TAGs, along with a limited number of glycoceramides present in the control group, were conspicuously absent in the MAD group. Conversely, a multitude of ceramides and phosphatidylethanolamines detected within the MAD group remained undetected in the control group. These findings indicate that the modified Atkins diet elicited specific and discernible alterations in lipid profiles. The compilation of unique metabolites identified within each respective group can be found in the **Supplementary Materials**.

## 4 Discussion

Safe and effective therapies for mitigating memory loss in elderly people with MCI and mild dementia due to AD are severely lacking. New therapies like lecanemab, an IgG1 monoclonal antibody shown to reduce markers of amyloid in the brains of patients with early AD (67), are not without limitations. Serious concerns have arisen regarding safety and cost as well as translation to meaningful clinical outcomes (5, 6). One promising strategy for treating this progressive neurodegenerative disease is to compensate for the reduced cerebral glucose utilization in AD (28, 68) using ketone bodies as an alternative energy source. In this study, we examined the feasibility of measuring changes in cognition and the lipids and metabolites during 12 weeks of use of a ketogenic diet therapy (MAD) compared to a recommended diet for elderly individuals.

The present study demonstrated that recruitment for dietary interventions in older adults with cognitive impairment is very challenging due to participant eligibility and interest. This study had high attrition after baseline but retained all participants who were in the study at week 6, which suggests a run-in period could greatly reduce attrition after randomization. Rates of attrition were comparable across treatment groups and to those in similar studies (50, 69, 70). Possibilities for enhancing recruitment, adherence, and retention in future efficacy studies could include the following: adjusting inclusion/exclusion criteria to capture individuals with greater willingness to change their dietary habits; educating study staff and participants that remaining in the study is extremely valuable even if they do not adhere to the assigned diet; conducting more virtual assessments, whether by telephone, video, or messaging; making in-person visits less frequent, shorter, and less burdensome; using automatic/scheduled reminders for adherence to study protocol at home; including incentives for adherence; and incorporating virtual consulting/coaching with a dietitian or nutritionist to supervise shopping and meal planning/preparation.

Pilot studies are often not powered to detect meaningful differences in the primary outcome with high confidence, but they can provide useful information to investigators planning future studies in this setting. While the estimates of the effect size and treatment effect on the change in MCS were positive and the majority of their 95% confidence intervals were greater than zero, they were not statistically significant. Results of cognitive outcome analyses should also be cautiously interpreted, given the issues of attrition, adherence, and influential observation.

Following a 12-week MAD intervention, we noted a significant decrease in circulating TAGs (50:5, 52:5, and 52:6). Additionally, certain TAGs (as indicated in **Table 3**) that were present in the control diet group were not detected in the ketogenic diet group. This suggests an enhanced breakdown of TAGs into fatty acids, likely driven by increased lipolysis, and their subsequent utilization through β-oxidation to produce energy and ketone bodies (61, 62). This interpretation gains support from the observation of elevated levels of ketone body (acetoacetate) production as well as metabolic byproducts from medium-chain fatty acids, including 3-hydroxyoctanoate and 5-hydroxyhexanoate. Moreover, in the MAD group, lower levels of fatty acylcarnitines, specifically hexanoylcarnitine and stearoylcarnitine, which are intermediates of fatty acid oxidation, were observed when compared to the control diet group. These collective findings lend further credence to the notion that the ketogenic diet prompts an increased utilization of fatty acids for energy production through β-oxidation, ultimately leading to the production of ketone bodies.

Both acetoacetate and medium-chain hydroxy fatty acids have been linked to potential cognitive benefits (71, 72). Acetoacetate, a type of ketone body produced during fatty acid metabolism, is suggested to have neuroprotective properties and is considered a potential alternative fuel source for the brain (71, 73). Some research suggests that ketone bodies like acetoacetate may offer neuroprotection and could be beneficial for cognitive function, especially in conditions like neurodegenerative diseases and certain brain injuries (73). However, the exact mechanisms and extent of cognitive improvement are still areas of ongoing research. Medium-chain hydroxy fatty acids, such as 3-hydroxyoctanoate and 5-hydroxyhexanoate, are derived from medium-chain fatty acids and are involved in energy metabolism (72). While the impact of specific hydroxy fatty acids in relation to cognitive outcomes remains under investigation, medium-chain fatty acids, in general, have been studied for their potential cognitive benefits (72). Some studies have suggested that the consumption of MCTs, which can produce medium-chain fatty acids and their derivatives like hydroxy fatty acids, might have positive effects on cognitive function, particularly memory, and brain health (72, 74–76). It is important to note that while there is emerging evidence supporting the potential cognitive benefits of acetoacetate and certain hydroxy fatty acids, the research is still in its early stages, and more studies are needed to establish the exact impact and mechanisms of these compounds on cognitive outcomes.

This study had several important limitations. First, sample sizes were small in both study groups, with challenges in recruitment, retention, and adherence (53). Dropout and adherence may be related to participant characteristics that are directly or indirectly related to their cognitive function, which can lead to selection bias. Second, although great care was taken to enhance the probability that participants’ cognitive impairments were due to AD, it is possible that some participants did not have the pathophysiology of AD, rendering the potential benefits of ketosis less likely. Third, participants, study partners, dieticians, and study coordinators were not blinded to the assigned diet, so the potential for bias cannot be entirely ruled out. In addition, the study’s eligibility criteria intentionally resulted in a sample free of comorbidities that are common in the elderly, including cardiovascular disease and severe diabetes. As such, results from this study may not be generalizable to the larger AD population, in which these comorbidities are also common. The study site and inclusion/exclusion criteria yielded a sample limited to mostly white and highly educated participants, which greatly limits the generalizability of our findings. We targeted patients with MCI and mild dementia due to AD. Perhaps even these early-stage patients are too advanced to benefit from a ketogenic diet. Implementing MAD in less cognitively impaired persons with “subjective cognitive decline” (77–81) might result in larger effects. Finally, not all of the participants who completed the study provided blood samples deemed adequate for analysis, primarily due to nonfasting and/or limited blood supply, which may have introduced bias as well.

## Data availability statement

The raw data supporting the conclusions of this article will be made available by the authors, without undue reservation.

## Ethics statement

The studies involving humans were approved by The Institutional Review Board of the Johns Hopkins University School of Medicine. The studies were conducted in accordance with the local legislation and institutional requirements. The participants provided their written informed consent to participate in this study.

## Author contributions

All authors listed have made a substantial, direct, and intellectual contribution to the work, and approved it for publication.
