# Supplementary material for: A randomized feasibility trial of the modified Atkins diet in older adults with mild cognitive impairment due to Alzheimer’s disease
Source: Front Endocrinol (Lausanne). 2024 Mar 4;15:1182519. doi: 10.3389/fendo.2024.1182519 (PMC10949529; doi:10.3389/fendo.2024.1182519)
Supplement: Supplementary file 1 [file Table_1.docx]

Supplementary Table 1. Eligibility criteria for participation in the trial.

| **Inclusion criteria:** |
| --- |
| 1. Age 60 years old or older |
| 2. Diagnosed with mild cognitive impairment (any subtype) or early Alzheimer’s disease by standard clinical criteria [[87](#_ENREF_87), [88](#_ENREF_88)] by a physician or neuropsychologist using all available information |
| 3. Clinical Dementia Rating (CDR) global score of 0.5 or 1 |
| 4. Montreal Cognitive Exam Score between 18 and 25 |
| 5. Residing with a cognitively-normal person willing to serve as study partner |
| 6. Approval for participation by primary care physician |
|  |
| **Exclusion criteria:** |
| 1. Unstable metabolic condition (documented on screening laboratory studies performed within the past year) |
| - 1. persistent hyponatremia (sodium < 130 mg/dL twice within the past year) |
| - 1. severe hypernatremia (sodium > 150 mg/dL twice within the past year) |
| - 1. hypoglycemia (glucose < 50 mg/dL) |
| - 1. hypocalcemia (albumin corrected calcium < 8 mg/dL) |
| 1. Type-I diabetes |
| 1. Type-2 diabetes requiring any medication other than metformin |
| 1. Liver failure |
| - 1. AST, ALT, or ammonia > 5x upper limits of normal |
| - 1. hyperbilirubinemia |
| - 1. total bilirubin > 15 mg/dL |
| - 1. direct bilirubin > 5 mg/dL |
| 1. Hypercholesterolemia (on medication, if needed) |
| - 1. fasting total cholesterol > 300 mg/dL |
| - 1. fasting LDL cholesterol > 200 mg/dL |
| 1. Known fatty acid oxidation disorder or pyruvate carboxylase deficiency |
| 1. Body mass index < 18.5 |
| 1. History of ischemic or hemorrhagic stroke |
| 1. History of nephrolithiasis |
| 1. History of myocardial infarction or known coronary artery disease |
| 1. Acute pancreatitis |
| 1. Any other concerns about nutritional status (e.g., recent unexplained weight loss, difficulty swallowing) |

Supplementary Table 2. Baseline demographic and clinical factors for participants with and without blood samples.

|  | **NIA** | | **MAD** | | **Overall** | |
| --- | --- | --- | --- | --- | --- | --- |
|  | **Does not have adequate blood samples (N=12)** | **Has adequate blood samples (N=6)** | **Does not have adequate blood samples (N=11)** | **Has adequate blood samples (N=9)** | **Does not have adequate blood samples (N=23)** | **Has adequate blood samples (N=15)** |
| **Age (Years)** |  |  |  |  |  |  |
| Mean (SD) | 76.3 (5.1) | 70.1 (5.3) | 75.6 (6.5) | 72.6 (5.2) | 75.9 (5.7) | 71.6 (5.2) |
| Range | [70.4, 83.9] | [60.9, 76.1] | [67.6, 87.1] | [64.9, 82.9] | [67.6, 87.1] | [60.9, 82.9] |
| **Sex** |  |  |  |  |  |  |
| 1. Male | 5 (41.7%) | 2 (33.3%) | 5 (45.5%) | 8 (88.9%) | 10 (43.5%) | 10 (66.7%) |
| 2. Female | 7 (58.3%) | 4 (66.7%) | 6 (54.5%) | 1 (11.1%) | 13 (56.5%) | 5 (33.3%) |
| **Race** |  |  |  |  |  |  |
| 1. White | 9 (75.0%) | 5 (83.3%) | 10 (90.9%) | 9 (100%) | 19 (82.6%) | 14 (93.3%) |
| 2. Non-white | 3 (25.0%) | 1 (16.7%) | 1 (9.1%) | 0 (0%) | 4 (17.4%) | 1 (6.7%) |
| **Education (Years)** |  |  |  |  |  |  |
| Mean (SD) | 15.5 (3.9) | 16.7 (2.7) | 15.7 (2.8) | 16.6 (1.1) | 15.6 (3.3) | 16.6 (1.8) |
| Range | [9, 20] | [12, 20] | [13, 20] | [16, 19] | [9, 20] | [12, 20] |
| **BMI** |  |  |  |  |  |  |
| Mean (SD) | 24.8 (2.8) | 29.1 (6.1) | 29.9 (5.8) | 26 (3.1) | 27.2 (5.1) | 27.3 (4.6) |
| Range | [17, 27.4] | [23.5, 39.7] | [18.6, 39.9] | [21.4, 31.2] | [17, 39.9] | [21.4, 39.7] |
| **MCS (HVLT + BVMT Delayed)** |  |  |  |  |  |  |
| Mean (SD) | 2.3 (3.1) | 4.5 (5.2) | 3.5 (4.3) | 6.9 (6.6) | 2.9 (3.7) | 5.9 (6) |
| Range | [0, 9] | [0, 12] | [0, 11] | [0, 17] | [0, 11] | [0, 17] |
| Missing | 1 (8.3%) | 0 (0%) | 0 (0%) | 0 (0%) | 1 (4.3%) | 0 (0%) |
| **MMSE-2 EV** |  |  |  |  |  |  |
| Mean (SD) | 35.5 (10) | 48.3 (12.6) | 40.5 (7.7) | 43.7 (11.5) | 38 (9.1) | 45.5 (11.8) |
| Range | [17, 51] | [23, 56] | [25, 50] | [29, 62] | [17, 51] | [23, 62] |
| Missing | 1 (8.3%) | 0 (0%) | 0 (0%) | 0 (0%) | 1 (4.3%) | 0 (0%) |
| **CDR Global Score** |  |  |  |  |  |  |
| Mean (SD) | 0.5 (0.1) | 0.7 (0.3) | 0.6 (0.2) | 0.6 (0.2) | 0.6 (0.2) | 0.6 (0.2) |
| Range | [0.5, 1] | [0.5, 1] | [0.5, 1] | [0.5, 1] | [0.5, 1] | [0.5, 1] |
